# Venovenous Extracorporeal Membrane Oxygenation Usage Following Bullet Embolism to the Pulmonary Artery

**DOI:** 10.31486/toj.23.0027

**Published:** 2024

**Authors:** Jonathan E. Schoen, Brian Carr, Murtuza Ali, Brett Chapman, Alan Marr, Lance Stuke, Patrick Greiffenstein, John P. Hunt, Paige Deville, Alison Smith

**Affiliations:** ^1^ Department of Surgery, Louisiana State University Health Sciences Center–New Orleans School of Medicine, New Orleans, LA; ^2^ Department of Medicine, Louisiana State University Health Sciences Center–New Orleans School of Medicine, New Orleans, LA; ^3^ Department of Surgery, Louisiana State University Health Sciences Center–Shreveport School of Medicine, Shreveport, LA

**Keywords:** *Extracorporeal membrane oxygenation*, *pulmonary embolism*, *respiratory distress syndrome*, *wounds–gunshot*

## Abstract

**Background:** Pulmonary artery embolus is a rare complication following gunshot wounds that creates a unique and serious challenge for trauma surgeons. While the majority of bullets that embolize through the vascular system end in the peripheral circulation, approximately one-third enter the central venous circulation.

**Case Report:** We present the case of a bullet embolus to the left pulmonary artery following gunshot wounds to the right chest and the abdomen, with the abdominal ballistic traversing the liver before entering the vena cava and embolizing. The patient's course was complicated by the development of severe acute respiratory distress syndrome that was successfully managed by venovenous extracorporeal membrane oxygenation.

**Conclusion:** Venovenous extracorporeal membrane oxygenation support for severe acute respiratory distress syndrome after bullet embolization to the pulmonary tree and surgical embolectomy is a viable option in appropriately selected patients.

## INTRODUCTION

Pulmonary artery embolus is a rare complication following gunshot wounds that creates a unique and serious challenge for trauma surgeons. While the majority of bullets that embolize through the vascular system end in the peripheral circulation, approximately one-third enter the central venous circulation.^1^ A 2018 systematic review focusing on bullet emboli to the heart and great vessels found that approximately 65% of bullets lodged in the right heart, while 32% were found in the pulmonary arterial tree.^2^ Despite increasing use of less invasive endovascular retrieval techniques, patients undergoing pulmonary embolectomy are still at risk for significant postoperative complications. We present the case of a bullet embolus to the left pulmonary artery following gunshot wounds to the right thoracic cavity and abdomen complicated by the development of severe acute respiratory distress syndrome (ARDS) that was managed by venovenous (VV) extracorporeal membrane oxygenation (ECMO).

## CASE REPORT

**Figure 1.**
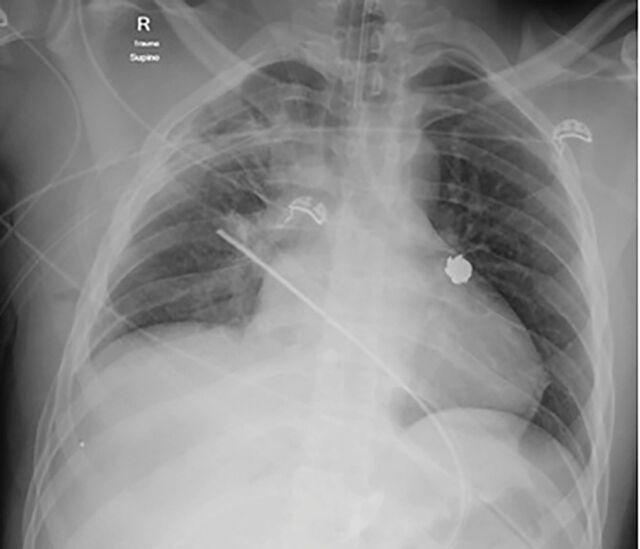
Initial chest x-ray showed a fluid-filled right chest with a bullet projecting over the left lung hilum.

**Figure 2.**
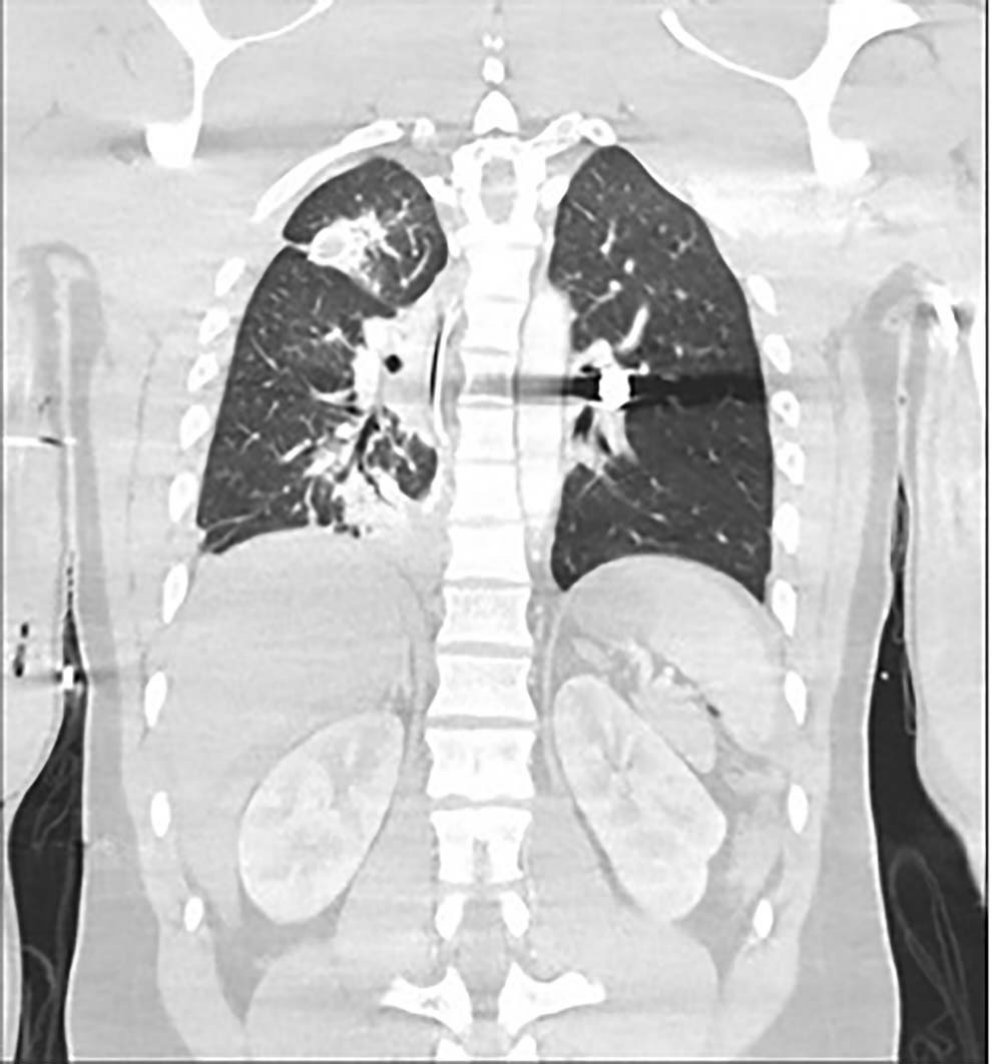
Preoperative computed tomography scan showed the bullet in the left pulmonary artery.

A 22-year-old male presented as a transfer from an outside facility for multiple gunshot wounds. En route, he was intubated, had a right chest tube placed, and received 6 units of packed red blood cells and 1 unit of fresh frozen plasma. Upon arrival at the hospital, he was tachycardic and normotensive. He had multiple gunshot wounds to his extremities, right upper quadrant, and right chest. Chest x-ray showed a fluid-filled right chest with a bullet projecting over the left lung hilum (Figure 1). Computed tomography scan confirmed the bullet in the left hilum without any clear ballistic tract (Figure 2). The patient also had a grade IV liver laceration with extension into the inferior vena cava and hemoperitoneum.

In the operating room, a left chest tube was placed with minimal output. A pericardial window was equivocal for bleeding. Exploratory laparotomy revealed a right diaphragm injury, a segment VII liver injury, a cecal injury, and a retroperitoneal zone III hematoma with active bleeding from the right psoas muscle. These injuries were addressed in a damage-control fashion with ileocecectomy, packing of the psoas and liver injuries for hemostasis, and temporary abdominal closure. Left thoracotomy revealed no traumatic violation of the pericardium. During evaluation of the left lung, a ballistic fragment was palpable within the hilum. The decision was made to leave the bullet in place and plan for an endovascular retrieval. Esophagogastroduodenoscopy showed no evidence of esophageal injury. The patient was taken to the intensive care unit (ICU) postoperatively for further resuscitation.

The patient subsequently underwent definitive operative management of his abdominal injuries: removal of hemostatic packing, creation of an ileocolonic anastomosis, and abdominal closure. Endovascular retrieval of the bullet with interventional radiology was pursued, with a plan for femoral venous cutdown for extraction. On preoperative workup, however, the bullet was noted to measure 14 mm while the patient's iliac veins measured 11 mm bilaterally, precluding endovascular retrieval. The decision was made to proceed with open retrieval of the bullet, and cardiothoracic surgery was consulted. Given the patient's recent laparotomy, transabdominal caval access with retrieval via endovascular means was considered, but given the ballistic injury and grade IV liver injury involving his vena cava, reopening the thoracotomy and performing a direct pulmonary artery embolectomy were thought to involve less risk.

**Figure 3.**
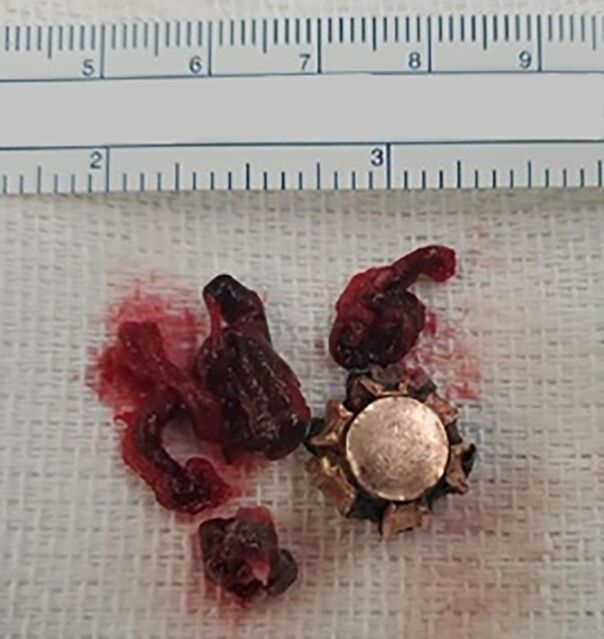
The retrieved bullet and associated clot burden.

The patient underwent open retrieval of the embolized bullet on hospital day 4. Through the previous left thoracotomy, the lung was mobilized, and the bullet was located within the inferior branch of the left pulmonary artery. On inspection, the bullet appeared to be eroding into the vessel wall. Proximal and distal control were obtained at the left main pulmonary artery and left inferior pulmonary artery, respectively, and an arteriotomy was performed. The bullet and a significant associated clot burden were retrieved (Figure 3), and the arteriotomy was closed. A 32 French (F) chest tube was placed, and the thoracotomy was closed. The patient received 2 units of packed red blood cells intraoperatively and was returned to the ICU.

Postoperatively, the patient had a significant decline in respiratory status, requiring positive end-expiratory pressure (PEEP) of 15 mm H_2_O and fraction of inspired oxygen (FIO_2_) of 100%. On hospital day 5, the patient had a Pao_2_/FIO_2_ of 64 mm Hg and lung compliance of 94.7 mL/cm H_2_O. Chest x-ray showed worsening left lung opacities and consolidation.

Therapeutic heparin was started, considering the clot burden noted in the pulmonary vasculature during bullet retrieval. The patient's Murray score was 3, and the ECMO team was consulted for evaluation. The patient was cannulated for VV ECMO using a dual lumen 31F right internal jugular ProtekDuo cannula (LivaNova, PLC) in a right-atrium-to-pulmonary-artery configuration. After initiation, the patient was placed on pressure-controlled ventilation in the 10-10-10-30% fashion (respiratory rate-inspiratory pressure-PEEP-FIO_2_%). Because of the patient's poor lung compliance, this pressure-controlled ventilation only delivered a tidal volume range of 2 to 4 cc/kg of ideal body weight, placing him in the ultra-lung-protective ventilation category.

The ECMO run was complicated by hypercoagulability issues. On circuit day 2, a 60% reduction in oxygenator efficiency was shown by postoxygenator PO_2_. On evaluation of the oxygenator, a large piece of fibrin clot was found in the outflow channel of the pump, and a significant clot burden was visible in the oxygenator. Both were exchanged. The patient's heparin anti-Xa goals were increased from the protocol standard of 0.3 to 0.5 IU/mL to 0.7 IU/mL. The patient required large doses of heparin to achieve this goal, reaching 96,000 units of unfractionated intravenous heparin on his first therapeutic day. Conversion to another anticoagulation method was considered. However, when thromboelastography demonstrated high functional fibrinogen levels, we decided against changing anticoagulants and instead to increase his heparin dose. On circuit day 3, the patient had a decrease in oxygenator efficiency, presumed to be secondary to further clot within the oxygenator. Because of decreased efficiency and decreased Pao_2_ despite high pump flows, the sweep rate was increased to 7 L/min. Oxygenation improved after this adjustment. The patient continued to have unmeasurably high fibrinogen levels. Alternative anticoagulation was again considered; however, the increased anti-Xa goals allowed no further propagation of clot or significant loss of oxygenator efficiency.

A sweep rate of 7 L/min was continued until circuit day 5. At that time, weaning was initiated with tidal volume increasing to standard-lung-protective ventilation parameters of 4 to 6 cc/kg of ideal body weight. Sweep and flow were weaned as the lungs recovered gas exchange capacity. Improved hemodynamics and oxygen delivery allowed aggressive diuresis. Successful decannulation was effected on hospital day 13. Total circuit time was less than 8 days.

The patient was extubated on hospital day 14. His subsequent course was complicated by *Bacteroides* empyema, which was treated with antibiotics, and bilateral pulmonary emboli, which were treated with continuous heparin and oral apixaban. The patient was discharged home on hospital day 44 with no supplemental oxygen requirement.

Clinic evaluation 4 weeks postdischarge was unremarkable. Two-year follow-up revealed that the patient was doing well with no significant limitations.

## DISCUSSION

Strategies continue to evolve for treatment of bullet embolism in trauma patients, but operative retrieval remains the standard procedure. The surgical procedure is high-risk with a not insignificant potential for perioperative complications. Endovascular retrieval is less invasive but is limited by factors such as facility capabilities and, as in this case, size mismatch between the bullet and the vasculature.

Pulmonary complications remain a significant risk regardless of which technique is used, and ARDS is a persistent major concern. While improvements in ARDS care have decreased the incidence and severity in trauma patients,^3^ a significant mortality risk remains in severe cases.

VV ECMO has shown promise in decreasing mortality in trauma patients with severe ARDS. When the CESAR trial was published in 2006, the results demonstrated improved survival and quality of life with ECMO in patients with ARDS.^4^ Since then, ECMO use in patients with ARDS has increased and has continued to improve survival.^5^

Hemorrhagic complications are a significant concern when using VV ECMO in trauma patients because of the necessary anticoagulation levels. Bleeding complications of any severity occur in 32.8% of cases, while major bleeding occurs in 27.9%.^6^ However, ECMO patients have a higher survival rate when anticoagulated with heparin vs no anticoagulation.^7^ Thrombosis is also a significant concern, as a subset of patients develops fibrinolytic shutdown in response to injury.^8^ Elevated fibrinogen levels and the associated decrease in fibrinolytic activity in trauma patients may increase the risk of circuit thrombosis.^9^ Thrombosis poses a threat to circuit efficiency and may necessitate circuit component exchange.

In our patient, the intrahepatic bullet trajectory through the vena cava and his profoundly prothrombotic state likely conferred a protective effect from a life-threatening hemorrhage. These injuries were concerning for anticoagulation on ECMO. However, no bleeding complications had occurred to that point while the patient had been anticoagulated for pulmonary embolic events, and the patient's mortality risk from ARDS was considerable without ECMO. We believed the benefit outweighed the risk.

## CONCLUSION

Pulmonary artery bullet embolism is a rare complication of gunshot wounds, and in such cases, pulmonary complications such as ARDS are significant. VV ECMO is a viable option to improve survival in trauma patients who develop severe ARDS postoperatively.

## Notes

*This article meets the Accreditation Council for Graduate Medical Education and the American Board of Medical Specialties Maintenance of Certification competencies for Patient Care and Medical Knowledge.*
